# Clinical Presentation and Management of Acute Dystonia from Drug Abuse or Misuse in Adolescents and Young Adults: A Retrospective Cohort Study in Bangkok, Thailand

**DOI:** 10.1155/2023/2725974

**Published:** 2023-04-04

**Authors:** Pootipong Wongveerasin, Rittirak Othong, Akkasil Pinchumponsang, Warunya Hungspruke, Peerarin Jongjaroenwit

**Affiliations:** Department of Emergency Medicine, Faculty of Medicine Vajira Hospital, Navamindradhiraj University, Bangkok, Thailand

## Abstract

**Objectives:**

To describe the clinical presentation of acute dystonia (AD) from drug abuse or misuse, as well as the emergency department (ED) management and outcomes in adolescents and young adults.

**Methods:**

This was a retrospective cohort study of patients aged 10–25 years who were admitted to the ED for AD due to intentional abuse or misuse from January 1, 2014, to June 30, 2017. Data were collected from electronic medical records by three investigators with excellent interrater reliability (0.87).

**Results:**

Sixty-two cases met the criteria with male predominance (85.5%); the mean age was 16.7 years. Perphenazine was the most common cause of AD (38.7%), followed by haloperidol (32.2%). The most common AD manifestations were torticollis (51.6%), oromandibular dystonia (45.2%), and oculogyric crisis (22.6%). Intravenous (IV) diazepam combined with oral trihexyphenidyl and IV diazepam alone were the most frequently used first treatment in our ED (41.7% and 35.0%, respectively). Overall, the improvement rates from IV diazepam alone or combined with trihexyphenidyl ranged from 46.2%–75.0%. These rates were inferior to those observed with IV benztropine (100%) alone or combined with trihexyphenidyl. All patients were treated on an outpatient basis, except for one who was admitted to a pediatric ward.

**Conclusions:**

In recent years, drug-induced AD caused by intentional abuse among adolescents and young adults has become a concern in Thailand. The most common suspected drugs of abuse were first-generation antipsychotics, perphenazine, and haloperidol. The most effective treatment was benztropine.

## 1. Introduction

During 2014-2015, we observed an unprecedented number of adolescents and young adults presenting to our emergency department (ED) with acute dystonia (AD). They often reported having tried “some pills” obtained from their peers to obtain a pleasurable effect.

In clinical settings, AD often occurs as an adverse drug reaction induced by antipsychotic drugs, more frequently with high-potency typical agents, e.g., haloperidol and fluphenazine. Some of atypical agents such as olanzapine and quetiapine can also cause AD but with low incident rates [1]. Due to the higher efficacy and less undesirable side effects of the second-generation antipsychotics (SGAs) for modern psychiatric treatment, their availability has been increasing tremendously. Misuse and abuse of the SGA, such as quetiapine and olanzapine, have also become more common [2]. In contrast, only a few pieces of literature on first-generation antipsychotic abuse have been published, mostly as case reports and before 1990 [3–5]. In addition to antipsychotics as a common cause of AD in the pediatric population, another most commonly implicated drug is an antiemetic [6, 7]. However, in Nigeria, the use of promethazine and antimalarial drugs is more common as a cause of AD [8]. Other drugs with dopamine receptor antagonist properties include anticonvulsants (carbamazepine and phenytoin), and a few stimulants (cocaine and MDMA) have been reported to cause AD [9].

Only a limited number of studies have investigated the clinical findings and ED management of drug-induced AD [6–8], and these studies reported drug-induced AD from therapeutic uses or accidental ingestion. However, the present study focused on AD from drug abuse or misuse among adolescents and young adults and describing the clinical presentation, ED management, and outcomes of the aforementioned population.

## 2. Methods

This retrospective cohort study was approved by the Ethics Committee of Vajira Hospital, and the data from January 1, 2014, to June 30, 2017, were collected from the electronic medical records of Vajira Hospital, Bangkok, Thailand. The hospital is a university hospital with 875 beds. Our emergency department has approximately 50,000 visits per year (140 visits a day). We included adolescents and young adults aged 10–25 years who were diagnosed with intentional abuse- or misuse-related AD. This age range of adolescents and young adults was defined by the Association of Maternal and Child Health Programs [10]. The term “intentional abuse” was defined as “an exposure resulting from the intentional improper or incorrect use where the patient was likely attempting to gain a high, euphoric effect or some other psychotropic effect, including recreational use of a substance for any effect” [11]. The term “intentional misuse” was defined as “an exposure resulting from the intentional improper or incorrect use for reasons other than the pursuit of a psychotropic effect” [11]. Patients with symptoms of AD that occurred within 7 days [1] since the last exposure via any route of at least one suspected drug were included in this study. We excluded those who took medications due to medically justifiable reasons and those with primary dystonia or secondary dystonia from other diseases, e.g., viral encephalitis and Wilson's disease. The inclusion and exclusion criteria are illustrated in Supplementary Figure 1.

We collected the following data: age, sex, history of ingestion of AD-inducing drugs, laboratory test results of blood chemistry and complete blood counts, ED treatment, duration of ED and hospital stay, and ED revisit within one week after ED discharge.

The characteristics of drugs reported by those patients are provided in Table 1. These agents were identified using databases of drug lists available in Thailand [12–14].

In a few cases, urine samples were sent for confirmatory comprehensive drug screening through liquid chromatography-tandem mass spectrometry, quadrupole time-of-flight (LC/MS/MS, Q-TOF).

For the treatment of each patient received, each cycle of treatment was separated by complete resolution of AD, followed by relapse. Each cycle of treatment involved monotherapy or a combination of medications; for example, the first treatment could involve the intravenous (IV) administration of diazepam, followed by oral trihexyphenidyl. We also examined the rate of ED revisit within 1 week after discharge.

Data extraction was performed by three investigators using a standardized data collection form and protocol. The three investigators were trained to collect data on ten mock medical records, and then, interrater reliability (kappa) was tested from the data extracted from ten actual medical records. The kappa scores ranged from 0.83 to 0.89 (mean: 0.87), indicating excellent interrater reliability. To minimize selection bias, those three investigators were blinded to the objectives of the study, as suggested by Gearing et al. [15]. Lastly, all collected data were reviewed and verified by an expert board-certified medical toxicologist.

## 3. Results

During the study period (Supplementary Figure 1), 62 cases were included in this study. The number of abuse and misuse cases was 61 cases and 1 case, respectively. Among them, the majority were male (Table 2). All patients had unremarkable medical history, except for three patients with underlying diseases. Almost all patients (except for two) had symptoms of AD upon arrival to the ED. Four patients reported a previous episode of drug-induced AD. All the drugs were taken via the oral route.

**Figure 1:**
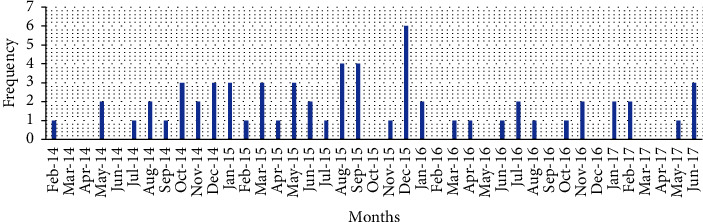
Number of cases recorded per month.

The monthly average number of cases throughout the entire study period of 3.5 years was 1.5 cases. In 2015, there was a four-fold increase in the number of cases versus the average of the entire study period (Figure 1). Our original intention was to begin case inclusion in 2013; however, after record search, we found no cases with drug abuse-induced AD in 2013 at all. The first case was discovered in February 2014.

As shown in Table 3, the most common suspected drug ingested was perphenazine (*n* = 25; 40.3%), followed by haloperidol (*n* = 23; 37.1%) and promethazine (*n* = 8; 12.9%). The onset of AD symptoms after drug ingestion was calculated based on data of 60 cases, yielding a mean of 27.2 ± 17.1 h. The most common drug of abuse as coingestion (i.e., no conclusive evident for causing AD) was tramadol (*n* = 25; 40.3%).

Urine samples from three patients were sent for LC/MS/MS, Q-TOF confirming the causative agents of AD as follows:

- Index case 1 (taking “B5” white tablet): haloperidol, diazepam and its metabolites (nordiazepam, temazepam, and oxazepam), nicotine, cotinine, hydroxycotinine, tramadol, nortramadol, dinortramadol, chlorpheniramine, and norchlorpheniramine
- Index case 2 (taking “B5” white tablet): haloperidol, acetaminophen, and diphenhydramine
- Index case 3 (taking “white ATC”): haloperidol, cetirizine, trihexyphenidyl, caffeine, theophylline, theobromine (caffeine metabolite), diazepam and its metabolites (nordiazepam, temazepam, and oxazepam), 7-aminoclonazepam, 7-acetamidoclonazepam, drofenine, and fenfluramine

Trihexyphenidyl and/or diazepam and its metabolites were from the treatment received in the ED (index cases 1 and 3).

The vital signs of all cases are mostly within normal limits with a slightly elevated heart rate (Table 2). Neurological examination revealed that almost all patients had a normal Glasgow Coma Scale score, mental status, pupil size, normal deep tendon reflex, and negative ankle clonus. Of note, two patients had a Glasgow Coma Scale score of 14, and four patients had diaphoresis. No one had signs of antimuscarinic (dilated pupils, dry axilla, and hypoactive bowel sound).

The most common manifestation of AD was torticollis (*n* = 32; 51.6%), followed by oromandibular dystonia (*n* = 28; 45.2%), and oculogyric crisis (*n* = 14; 22.6%) (Table 2).

A total of 51 patients (81.0%) underwent blood laboratory examinations, and the results are shown in Table 4.

The patients were treated in the ED using one or a combination of the following medications: benztropine (2 mg intramuscularly or intravenously), diazepam (5–10 mg intravenously), and trihexyphenidyl (2–4 mg orally thrice daily or 5 mg orally twice daily).  Table 5 shows treatment outcomes according to the type of pharmacological treatment. Two patients did not receive medications. One of them no longer had any AD symptoms upon arrival at the ED and received supportive treatment. The other with torticollis was transferred to another hospital due to health insurance-related preference. One patient received oral trihexyphenidyl alone because of the presence of only mild oculogyric crisis. Another patient received oral trihexyphenidyl as a first treatment but relapsed while in the ED.

Interestingly, all patients treated with parenteral benztropine alone or in combination with other medications, regardless of the cycle of treatment, recovered completely without any relapses in the ED or revisited the ED within 1 week. Of note, IV diazepam alone (the most commonly used medication for drug-induced AD in our ED) in either of the three cycles of treatment was associated with success rates without relapses ranging from 46.2%–61.9%. When IV diazepam was combined with oral trihexyphenidyl, these rates ranged from 52%–75%. Two patients relapsed after receiving the third cycle of treatment; both received IV diazepam. After 13 h 11 min in the ED, one of them (treated with five doses of 5 mg IV diazepam + 4 mg oral trihexyphenidyl) was discharged. The other patient (14-year-old boy) spent 11 h 30 min in the ED and was subsequently admitted to a pediatric ward where he further received multiple doses of IV diazepam and oral trihexyphenidyl. However, he was discharged after 3 days in the hospital.

The ED triage-to-discharge time and treatment-to-discharge time among those who received the first cycle of treatment were 06:10 and 05:10 h, respectively.

Forty-five patients (72.6%) received trihexyphenidyl (6–12 mg/day for 1–5 days) as home medication. During the 7-day follow-up period, two cases revisited the ED due to relapse of AD. One of them (17-year-old male) left the ED early, against medical advice, after receiving a dose of IV diazepam and showing improvement, but trihexyphenidyl was not brought back home with him. Approximately 18 h later, he returned to the ED due to focal limb dystonia. The other patient (24-year-old male) received IV diazepam and showed improvement. He was observed in the ED for 6.5 h prior to discharge with trihexyphenidyl prescribed as home medication. However, he did not take trihexyphenidyl in the ED or at home. At 1.5 h after discharge from the ED, he returned to the ED with opisthotonos and oculogyric crisis.

## 4. Discussion

In the past years, antipsychotics have gained attention as potential drugs of abuse or misuse worldwide [16–18]. Most of these studies highlighted problems related to SGA, particularly quetiapine, which could cause several unwanted effects, but rarely results in AD [18]. The increasing frequency of SGA abuse mirrors the increasing rate of SGA use in psychiatric treatment. In contrast, the use of the typical or first-generation antipsychotics (FGAs) has markedly decreased [19]. This may explain the rarity of FGA abuse. Our literature search on PubMed for FGA abuse yielded few publications after 1990. Notably, the literature on FGA abuse before 1990 was composed of case reports or small case series [3–5]. This study contained a relatively large number of cases of FGA abuse.

Previous studies on drug-induced AD from Korea [7], Turkey [6], and Nigeria [8] were conducted in therapeutic settings in pediatric patients. In contrast, our study was performed in a drug abuse setting. Unlike in the aforementioned studies [6–8], our patients were predominantly male, which is more in line with a systematic review by van Harten et al. [1], which has demonstrated that males are more likely to develop AD than females. This could also be attributed to the prevalently male population linked to drug abuse settings [18]. Those previous studies [6–8] reported high rates of gastrointestinal medications as causative agents for AD, whereas antipsychotics were vastly more common in our study.

Interestingly, the physical examination data analyzed in our study did not show the antimuscarinic effects of promethazine [20]. Although haloperidol and perphenazine do not have antimuscarinic effects [21], patients taking these agents had a mean heart rate of approximately 100 bpm and mildly elevated blood pressure. These effects could be the results of acute dystonic reactions [22] rather than antimuscarinic effects, since patients did not have other signs of antimuscarinic effects (e.g., dilated pupils, dry skin, and absent bowel sound).

Diazepam (IV) was the first drug of choice in our ED since it is readily available, in addition, because diazepam (IV) and trihexyphenidyl (oral) are covered by the universal healthcare program of the government of Thailand and are inexpensive (diazepam: 4 Thai baht per vial (10 mg) and trihexyphenidyl (2 mg) 0.5 Thai baht per tablet (1 USD = 32–35 Thai baht)), whereas benztropine (IV) is not, and it is relatively expensive (1 vial = 400 Thai baht). In general, the onset of diazepam (IV) and benztropine (IV) action occurs within a few minutes [23, 24], while that of trihexyphenidyl (oral) is 60 min [25]. Moreover, the duration of IV diazepam and oral trihexyphenidyl action is 12 h and 6–12 h, respectively [23, 25]. Initially, at the beginning of the outbreak, we often experienced delay in treatment when we used benztropine as a first-line treatment. After investigating into the cause of the delay, we found that benztropine was not listed as an emergency medication; thus, it is not readily available in the ED. It had to be paid first at the hospital pharmacy. The problem occurred because many of patients' friends, who often encountered patients with AD symptoms and brought them to our ED, could not afford to pay for benztropine because of its price. Consequently, diazepam was the most common prescription for drug-induced AD in our ED at that time due to its availability and much lower costs.

We observed that IV diazepam, administered at an optimal dosage of 5–10 mg (0.1 mg/kg) for the treatment of AD [21], could resolve AD in a short period of time. Nevertheless, patients who received IV diazepam were associated with a higher rate of relapse than those who received IV benztropine (Table 5). Park et al. [7] found that benzodiazepines were as effective as anticholinergic drugs. In contrast, our data suggested that benztropine (an anticholinergic agent) was superior to diazepam (benzodiazepine) in treating drug-induced AD in terms of relapses. In fact, benztropine is an indirect-acting dopamine agonism, which can increase dopamine release. Benztropine is also one of the most potent dopamine reuptake inhibitors [26]. As a result, the action of benztropine is very direct and specific in treating drug-induced acute dystonia. We attempted to overcome this shortcoming by combining IV diazepam and oral trihexyphenidyl (diazepam needs to be injected first to terminate dystonic reactions before they could take trihexyphenidyl by mouth). This approach could balance the costs of treatment and efficacy. However, we still found relapses in patients treated with the combination of these two drugs. This was attributed to the nonadherence of patients to treatment (i.e., oral trihexyphenidyl was not taken by several patients in the ED after the administration of IV diazepam). Consequently, in the subsequent stage of the study period, we encouraged ED physicians to administer oral trihexyphenidyl after IV diazepam as soon as patients improved and could take oral medication, since the time-to-peak effect of oral trihexyphenidyl is approximately 2-3 h [25, 27]. Two patients who developed AD at home prior to ED arrival, which was then temporarily resolved in the ED, received only oral trihexyphenidyl without IV medication (neither IV diazepam nor benztropine) and relapsed while in the ED. This could be explained from the time-to-peak effect of its form, which requires 2-3 h. Since anticholinergics have shorter half-lives than antipsychotics [28], the oral form of anticholinergics should be taken as home medications for another 48–72 h to prevent the occurrence of relapses of drug-induced AD after discharge from the ED [6]. Derinoz successfully used parenteral diphenhydramine for the treatment of drug-induced AD [6]. Diphenhydramine injection form just became available in Thailand in 2015 via the Thai National Antidote Program funded by the National Health Security Office (NHSO) [29]. However, at that time, since it has just been available, it was not well known and so not widely used among Thai physicians. Intravenous diphenhydramine could be an excellent alternative to parenteral benztropine at a much lower price.

Finally, we tried to find risk factors for relapse; however, none of the following factors were associated with relapse either with univariate or multivariate analysis (Table 6): age, sex, triage level, history of abusing drug, number of drug abuse, and types of medication for the first treatment.

## 5. Limitations

This was a retrospective study, performed in a single center, with some missing data.

## 6. Conclusions

This study investigated drug-induced AD from drug abuse/misuse among adolescents and young adults. Abuse was much more common than misuse. The most common suspected drugs of abuse were perphenazine and haloperidol. Relapses of AD from drug abuse during ED stay were common, particularly in patients treated with IV diazepam, oral trihexyphenidyl, or both. Benztropine was more effective; none of the patients receiving this medication had relapses.

## Figures and Tables

**Table 1:** Drug characteristics reported by patients and corresponding drug identifications.

Drug description/street name	Drug identification
“Yellow ATC tablet,” “round yellow tablet”	Perphenazine 2 mg
“Green ATC tablet”	Perphenazine 4 mg
“Purple ATC tablet,” “purple tablet with a small triangle in the middle”	Perphenazine 8 mg
“Zolam”	Perphenazine 8 mg
“Pink round tablet”	Perphenazine 4, 8, or 16 mg
“ATC,” “B5”	Haloperidol
“White ATC tablet,” “white ATC”	Haloperidol 2, 5, 10 mg
Light blue round tablet with “a mark” on it, “light blue round tablet”	Haloperidol 5, 10 mg
“Roche,” “white round Roche tablet”	Unspecified

^*∗*^ATC is the name of a pharmaceutical company that manufactures the tablet, Atlantic Laboratories Corp, LTD.

**Table 2:** Patient characteristics.

Demographics	Number (*n* = 62)	(%)
Sex		
Male	53	85.5
Age (years)		
Mean (±standard deviation)	16.7 ± 3.0	
10–14 years	15	24.2
15–19 years	38	61.3
20–24 years	9	14.5
Past medical history		
No known underlying disease	59	95.2
HIV infection	1	1.6
Thrombocytopenia	1	1.6
Allergy	1	1.6
Past history of drug abuse		
First time	42	69.4
2-3 times	3	4.8
4-5 times	0	0.0
>5 times	3	3.2
No data	14	22.6
Single/multiple drug (s) use		
Single-drug use	23	37.1
Combination of two or more drugs	34	54.8
Missing data	5	8.1
Purpose of drug use		
Abuse	61	98.4
Misuse	1	1.6
Physical examination		
Vital signs (at ED triage)		
Body temperature (°C) (*n* = 49), mean (±S.D.)	36.7 ± 0.4	
Heart rate (bpm) (*n* = 62), mean (±S.D.)	100 ± 20	
Respiratory rate (/min) (*n* = 61), mean (±S.D.)	20 ± 4	
Blood pressure, mean (±S.D.)		
Systolic (mmHg) (*n* = 62)	135 ± 19	
Diastolic (mmHg) (*n* = 62)	80 ± 12	
Neurological examination		
Mental status (*n* = 62)		
Normal	57	91.9
Agitated	1	1.6
Drowsy	2	3.2
No data	2	3.2
Pupil size (mm)		
Right (*n* = 60), mean (±S.D.)	2.93 ± 0.61	
Left (*n* = 60), mean (±S.D.)	2.88 ± 0.72	
Skin sign (autonomic) (*n* = 40)		
Normal	36	90.0
Diaphoresis	4	10.0
Dry	0	0.0
Bowel sound (autonomic) (*n* = 58)		
Normoactive	56	96.6
Hyperactive	2	3.4
Hypoactive	0	0.0
Characteristics of acute dystonic reactions (there may be > 1 sign per case)	*n* = 62 (93 signs in total)	% (calculated from 62 cases)
Spasmodic torticollis	32	51.6
Oromandibular dystonia	28	45.2
Oculogyric crisis	14	22.6
Focal limb dystonia	11	17.7
Facial grimacing	4	6.5
Opisthotonos	3	4.8
Laryngeal dystonia	1	1.6

ED, emergency department; S.D., standard deviation.

**Table 3:** Suspected drugs ingested by patients with acute dystonia (AD) in this study.

Suspected drugs causing AD (*n* = 62)	Number	(%)
Perphenazine	24	38.7
Haloperidol	20	32.2
Promethazine	3	4.8
Risperidone (from misuse)	1	1.6
Haloperidol + promethazine	3	4.8
Perphenazine + promethazine	1	1.6
Promethazine + unspecified drug	1	1.6
Unspecified drug	4	6.5
Missing data	5	8.1
Reported drugs/substances of abuse as “coingestion”		
Tramadol	25	40.3
Antihistamines	9	14.5
(i) Chlorpheniramine	3	4.8
(ii) Cetirizine	3	4.8
(iii) Unspecified	2	3.2
(iv) Dimenhydrinate	1	1.6
Cough syrup, unspecified	3	4.8
Codeine	1	1.6
Kratom	1	1.6

**Table 4:** Clinical investigation.

Investigation	Mean (±standard deviation)
*Chemistry*	
Blood glucose (mmol/L) (*n* = 33)	7 ± 2.1
Sodium (mmol/L) (*n* = 51)	140.4 ± 2.3
Potassium (mmol/L) (*n* = 51)	4.1 ± 0.5
Chloride (mmol/L) (*n* = 51)	105.5 ± 3.6
Bicarbonate (mmol/L) (*n* = 51)	25.0 ± 3.0
Phosphate (mg/dL) (*n* = 30)	3.6 ± 0.8
Magnesium (mg/dL) (*n* = 32)	2.2 ± 0.3
Calcium (mg/dL) (*n* = 35)	9.6 ± 0.4
Albumin (g/L) (*n* = 27)	43 ± 9
Blood urea nitrogen (mg/dL) (*n* = 48)	10.1 ± 3.3
Creatinine (mg/dL) (*n* = 47)	1.1 ± 0.2
Creatine kinase (U/L) (*n* = 31)	200.0 ± 96.9

*Complete blood count*	
White blood cells (×10^9^ cells/L) (*n* = 47)	10.0 ± 3.0
Hematocrit (%) (*n* = 47)	42.3 ± 4.2
Platelet count (×10^9^ cells/L) (*n* = 47)	265.6 ± 89.8
Neutrophils (%) (*n* = 47)	71.3 ± 14.1

**Table 5:** Treatment and outcomes.

Treatment in the ED	Number (*n* = 62)	(%)
Treated once	39	62.9
Treated twice	13	21
Treated thrice	6	9.7
Treated >3 times	2^†^	3.2
No medication administered	2^‡^	3.2

First treatment	Number *n* = 60 (%)	Outcome
Diazepam (IV) + trihexyphenidyl (oral)	25 (41.7%)	Improved 13 (52%) Relapse 12 (48%)
Diazepam (IV)	21 (35%)	Improved 13 (61.9%) Relapse 8 (38.1%)
Benztropine (IV) + trihexyphenidyl (oral)	9 (15%)	Improved 9 (100%)
Benztropine (IV)	3 (5%)	Improved 3 (100%)
Trihexyphenidyl (oral)	2 (3.3%)	Improved 1 (50%) Relapse 1 (50%)

Second treatment	Number *n* = 21 (%)	Outcome
Benztropine (IV)	1 (4.8%)	Improved 1 (100%)
Diazepam (IV)	13 (61.9%)	Improved 6 (46.2%) Relapse 4 (30.7%) Non-responsive 3 (23.1%)
Trihexyphenidyl (oral)	1 (4.8%)	Improved 1 (100%)
Benztropine (IV) + trihexyphenidyl (oral)	1 (4.8%)	Improved 1 (100%)
Diazepam (IV) + benztropine (IV)	1 (4.8%)	Improved 1 (100%)
Diazepam (IV) + trihexyphenidyl (oral)	4 (19.0%)	Improved 3 (75.0%) Relapse 1 (25.0%)

Third treatment	Number *n* = 8 (%)	Outcome
Benztropine (IV)	1 (12.5%)	Improved 1 (100%)
Diazepam (IV)	2 (25.0%)	Improved 1 (50%) Relapse 1^†^ (50%)
Trihexyphenidyl (oral)	1 (12.5%)	Improved 1 (100%)
Diazepam (IV) + trihexyphenidyl (oral)	4 (50%)	Improved 3 (75%) Relapse 1^†^ (25%)

^†^These two patients received more than three cycles of treatment: one was admitted to the pediatric department. ^‡^Received supportive treatment or transferred to another hospital. ED, emergency department; IV, intravenous.

**Table 6:** Risk factors associated with the relapse of drug-induced acute dystonia.

	Univariate analysis		Multivariate analysis	
	OR (95% CI)	*p* value	aOR (95% CI)	*p* value
Ages <16 years old (*N* = 60)	1.27 (0.44–3.67)	0.67		
Male (*N* = 60)	2.05 (0.46–9.25)	0.35		
Triage level: ESI 2 (*N* = 60)	2.00 (0.44–8.99)	0.37		
First Rx: trihexyphenidyl (*N* = 60)	1.90 (0.11–32.00)	0.67		
First Rx: IV diazepam 5 mg (*N* = 60)	0.53 (0.15–1.91)	0.33		
First Rx: IV diazepam 10 mg (*N* = 60)	8.94 (0.93–86.09)	0.06	7.67 (0.67–88.00)	0.10
First Rx: IV diazepam 5 mg + oral trihexyphenidyl (*N* = 60)	1.38 (0.46–4.21)	0.57		
First Rx: IV diazepam 10 mg + oral trihexyphenidyl (*N* = 60)	8.94 (0.93–86.09)	0.06	10.25 (0.93–112.58)	0.06
History of abusing drug more than once (*N* = 48)	4.46 (0.72–27.51)	0.11	5.45 (0.81–36.80)	0.08
Abused multiple drugs (*N* = 56)	0.95 (0.31–1.92)	0.94		

Using logistic regression, multivariate models were developed by adjusting for covariates with *p* < 0.2 in univariate models. OR = odds ratio, aOR = adjusted odds ratio, ESI = emergency severity index, IV = intravenous, mg = milligrams.

## Data Availability

The data used to support the findings of this study are available from the corresponding author upon request.

## Supplementary Materials

Supplementary Materials Supplementary Figure 1 shows the inclusion and exclusion processes of this study.
