# Supplementary material for: Clinical Presentation and Management of Acute Dystonia from Drug Abuse or Misuse in Adolescents and Young Adults: A Retrospective Cohort Study in Bangkok, Thailand
Source: Emerg Med Int. 2023 Apr 4;2023:2725974. doi: 10.1155/2023/2725974 (PMC10089775; doi:10.1155/2023/2725974)
Supplement: Supplementary Materials — Supplementary Figure 1 shows the inclusion and exclusion processes of this study. [file 2725974.f1.docx]

**Supplementary Materials**

**Supplementary Figure 1: Inclusion and exclusion process**

**Patients with acute dystonia (AD) presented to the emergency department (ED) from January 1, 2014 to June 30, 2017 (n = 102)**

**Not meeting inclusion criteria**

**Excluded**

**c**

**Aged <10 or >25 years (n = 23)**

**History or suspicion of non-drug induced AD, at least once before presenting to or at the ED (n = 5)**

**Symptoms occurred >7 days since last ingestion of a substance (n = 1)**

**Medical records unavailable (n = 7)**

**AD occurred due to medications taken for medically justifiable reasons, rather than substance abuse (n=4)**

**A total of 62 cases were finally included**
